# Exosome Degeneration in Mesenchymal Stem Cells Derived from Patients with Type 1 Diabetes Mellitus

**DOI:** 10.3390/ijms222010906

**Published:** 2021-10-09

**Authors:** Michiko Horiguchi, Yuko Okada, Yuya Turudome, Kentaro Ushijima

**Affiliations:** Division of Pharmaceutics, Faculty of Pharmaceutical Sciences, Sanyo-Onoda City University, Sanyo Onoda 756-0884, Yamaguchi, Japan; mahoroba8172@icloud.com (Y.O.); tsurudome-y19@rs.socu.ac.jp (Y.T.)

**Keywords:** exosome, mesenchymal stem cells (MSCs), adipose-derived mesenchymal stem cells (ADSC), type 1 diabetes mellitus, CD9

## Abstract

Type 1 diabetes mellitus is characterized by the destruction of pancreatic β-cells and requires the regeneration of these destroyed pancreatic β-cells for radical treatment. The degeneration of organelles in stem cells compromises stem cell quality; however, organelles in the mesenchymal stem cells of patients with type 1 diabetes mellitus have not been characterized previously. In this study, we use transmission electron microscopy to evaluate the degeneration of organelles in adipose-derived stem cells of patients with type 1 diabetes mellitus (T1DM ADSCs). Compared to adipose-derived stem cells from healthy humans, T1DM ADSCs degenerate differently, characterized by prominent enlarged spherical vesicles. The exosomes of T1DM ADSCs are found to be enlarged, reduced in number, and increased in the percentage of those positive for tetraspanin CD9. The findings of this study provide insight into the characteristics of stem cells in patients with type 1 diabetes mellitus.

## 1. Introduction

Diabetes mellitus is a disease wherein carbohydrates, lipids, and proteins are metabolized aberrantly due to the insufficient secretion and action of insulin [1]. Diabetes mellitus can be divided into two major types: Type 1 diabetes mellitus, characterized by the destruction of pancreatic β-cells, and type 2 diabetes mellitus, characterized by impaired insulin secretion and insulin resistance [2,3,4]. In particular, patients with type 1 diabetes mellitus require insulin replacement therapy for life support and the regeneration of insulin-producing β-cells destroyed by autoantibodies and other factors for radical treatment [5].

Embryonic stem cells, induced pluripotent stem cells (iPSCs), and mesenchymal stem cells (MSCs) have been used in studies on regenerative therapy for diabetic patients [6,7,8]. MSCs have been reported to increase the efficacy of regenerative therapy by secreting growth factors, immune regulators, and tissue regeneration factors [9]. Among the MSCs obtained from various tissues, adipose-derived mesenchymal stem cells (ADSCs) and bone marrow-derived mesenchymal stem cells (BMSCs) have been studied for the regenerative treatment of various organs [10]. ADSCs have been reported to be more abundant in tissues and to produce greater amounts of vascular endothelial growth factor (VEGF) and hepatocyte growth factor (HGF) than BMSCs [11].

Recent reports have revealed the functions of various stem cells; however, the characteristics of stem cells found in pathological conditions remain largely unknown. We previously reported the characteristics and effects of transplantation of adipose-derived MSCs of patients with type 2 diabetes mellitus (T2DM ADSCs) [12]. We showed the degeneration of nuclei and mitochondria and reduced adenosine triphosphate (ATP) activity in T2DM ADSCs [12]. Furthermore, we reported that T2DM ADSCs have low transplantation efficiency, proliferative capacity, and stemness, and are prone to apoptotic cell death [13]. Moreover, we identified the forkhead box protein O (FoxO) signaling pathway-related genes glucose 6 phosphatase 3 (*G6PC3*) and insulin-like growth factor 1 (*IGF1*) as the genes responsible for transplantation resistance through a comprehensive mRNA analysis using next-generation sequencing technology [13]. Furthermore, we demonstrated that *G6PC3* and *IGF1* double-knockdown improves the transplantation efficiency of T2DM ADSCs [13].

However, the degeneration of organelles in T1DM ADSCs has not been investigated. In this study, we characterize organelles in adipose-derived stem cells of patients with type 1 diabetes mellitus (T1DM ADSCs) using a transmission electron microscopy (TEM).

## 2. Results

### 2.1. Degeneration of Organelles in Stem Cells Derived from Patients with Type 1 Diabetes Mellitus

**Figure 1:**
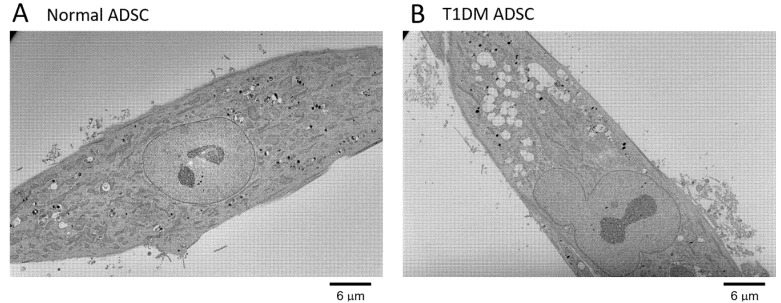
Electron microscopic images of the whole cells in adipose-derived mesenchymal stem cells (ADSCs). Whole-cell transmission electron microscope (TEM) images of ADSCs are shown. (**A**) A whole-cell image of normal ADSCs. (**B**) A whole-cell image of adipose-derived stem cells of patients with type 1 diabetes mellitus (T1DM ADSCs). The scale bar measures 6 µm.

**Figure 2:**
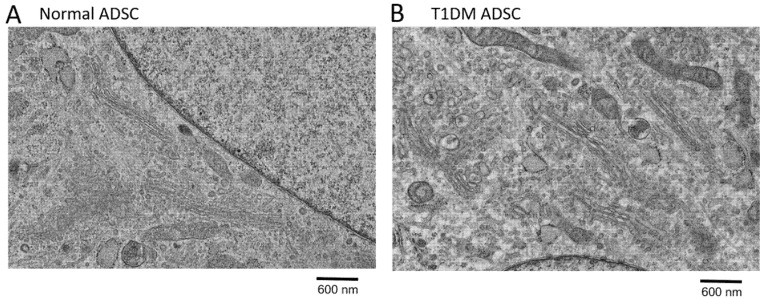
Electron microscopic images of the intracellular vesicles in adipose-derived mesenchymal stem cells (ADSCs). Transmission electron microscopic (TEM) images showing the morphology of the intracellular vesicles in ADSCs are shown. (**A**) An image of intracellular vesicles in normal ADSCs. (**B**) An image of intracellular vesicles in adipose-derived stem cells of patients with type 1 diabetes mellitus (T1DM ADSCs). The scale bar measures 600 nm.

This study aimed to characterize organellar changes in T1DM ADSCs. In this study, the degeneration of mitochondria and the nuclear membrane in T1DM ADSCs was evaluated using transmission electron microscopy. In T1DM ADSCs, no degenerated mitochondria or nuclear membranes were observed (Figure 1). However, the T1DM ADSCs had a greater number of spherical intracellular vesicles than the MSCs derived from healthy humans (normal ADSCs) (Figure 1). Magnified electron microscopic images of the intracellular vesicles in the ADSCs revealed that the spherical intracellular vesicles in the T1DM ADSCs were enlarged (Figure 2). These results showed that the degeneration in the T1DM ADSCs was distinct from that in the stem cells derived from patients with type 2 diabetes mellitus, and was characterized by prominently enlarged spherical intracellular vesicles.

### 2.2. Enlargement of Exosomes in Stem Cells Derived from Patients with Type 1 Diabetes Mellitus

**Figure 3:**
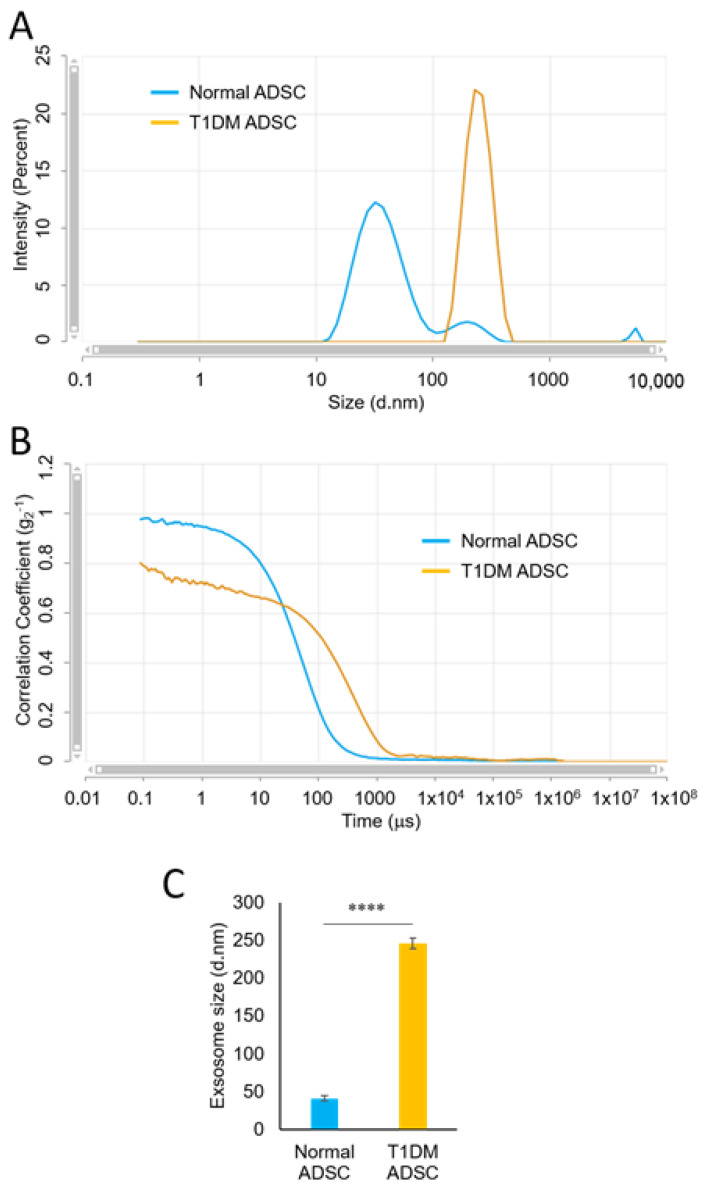
Exosome particle diameters of adipose-derived mesenchymal stem cells (ADSCs). Results of particle size measurements of ADSC exosomes. (**A**) A graph showing size distribution by intensity measured with a Zetasizer. (**B**) A correlogram generated using a Zetasizer. (**C**) Mean particle diameters of exosomes extracted from normal and adipose-derived stem cells of patients with type 1 diabetes mellitus (T1DM ADSCs). The values shown in the graph are in the form of mean ± SD. The number of samples is *n* = 5. Three independent evaluations were performed. Shapiro–Wilk and unpaired *t*-tests were used for statistical analysis. **** The *p*-value < 0.0001.

**Figure 4:**
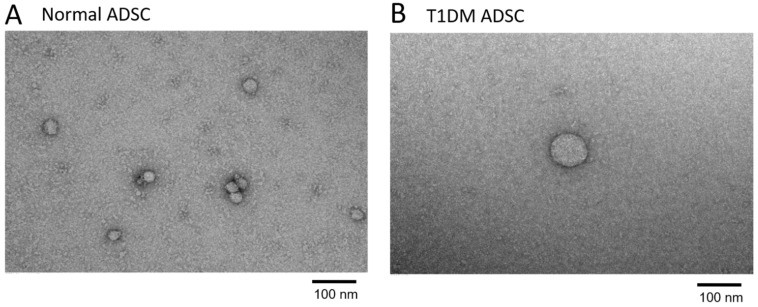
Electron microscopic images of exosomes. Transmission electron microscopic (TEM) images showing the morphology of exosomes extracted from adipose-derived mesenchymal stem cells (ADSCs). (**A**) An image of exosomes from normal ADSCs. (**B**) An image of exosomes from adipose-derived stem cells of patients with type 1 diabetes mellitus (T1DM ADSCs). The scale bar measures 100 nm.

Using a particle size distribution analyzer based on dynamic light scattering (DLS), we measured the diameters of the exosomes extracted from the ADSC culture supernatant. The fine particles dispersed in a suspension undergo Brownian motion; thus, larger particles move slow, and smaller particles move fast. When the particles undergoing Brownian motion are irradiated with laser light, the scattered light from the particles fluctuates according to the velocity of Brownian motion. In the DLS method, dispersed particles are irradiated with laser light, and the scattered light is observed with a photon detector. The laser intensity-based particle size analysis showed that the normal ADSCs had three peaks. The peak including the largest proportion of particles had a mean particle diameter of 38.4 nm (Figure 3A). Meanwhile, the T1DM ADSCs had one peak with a mean particle diameter of 252 nm. These results showed that the T1DM ADSC exosomes were larger in diameter than the normal ADSC exosomes (Figure 3A). The correlation coefficient in the mean particle diameter measurements is shown in the graph in Figure 3B. The mean exosome diameters from measurements with multiple independent samples were 40.84 ± 1.42 nm for the normal ADSCs and 245.80 ± 3.02 nm for the T1DM ADSCs, showing a significantly increased diameter of the T1DM ADSC exosomes (Figure 3C). Furthermore, the electron microscopic observation of the exosomes extracted from the T1DM ADSCs and those extracted from the normal ADSCs at the same magnification confirmed that the T1DM ADSC exosomes were larger than the normal ADSC exosomes (Figure 4).

### 2.3. A Reduced Quantity of Exosomes in Stem Cells Derived from Patients with Type 1 Diabetes Mellitus 

**Figure 5:**
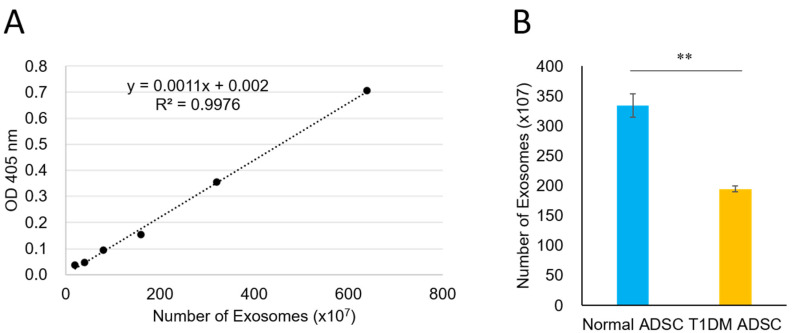
Quantification of exosomes for adipose-derived stem cells of patients with type 1 diabetes mellitus (T1DM ADSC). Results of quantification of exosomes extracted from adipose-derived mesenchymal stem cells (ADSCs) by colorimetry using acetylcholinesterase (AChE) activity as an index. (**A**) A standard curve created with two-fold serial dilutions of an exosome AChE standard. (**B**) A graph showing exosome counts calculated using the standard curve obtained from absorbance values measured at 405 nm by colorimetry; the exosomes were collected from the same volume of normal and T1DM ADSCs culture supernatants. Values shown in the graph are in the form of mean ± SEM. The number of samples is *n* = 3. Three independent evaluations were performed. Shapiro–Wilk and unpaired *t*-tests were used for statistical analysis. ** The *p*-value was 0.0023.

The electron microscopic images of the exosomes extracted from an equal volume of the T1DM ADSC or normal ADSC culture supernatant showed that the observed amount of T1DM ADSC exosomes was less than that of normal ADSC exosomes (Figure 4). Thus, we quantified the exosomes by colorimetry using acetylcholinesterase (AChE) activity as an index. The standard curve prepared using the standard material was y = 0.0011x + 0.002 (*R*^2^ = 0.9976) (Figure 5A). The exosome counts determined using the standard curve were (334.33 ± 19.36) × 10^7^ and (194.81 ± 5.34) × 10^7^ in 3 mL of culture supernatant for the normal ADSCs and the T1DM ADSCs, respectively, showing a significant decrease in the latter (Figure 5B).

### 2.4. An Increased Number of CD9-Positive Exosomes in Stem Cells Derived from Patients with Type 1 Diabetes Mellitus 

**Figure 6:**
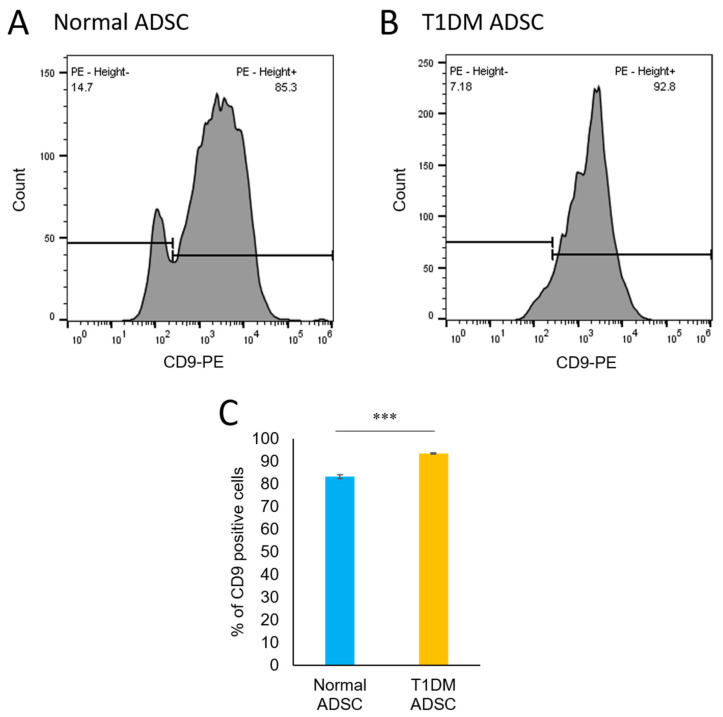
Characteristics evaluation of exosomes in stem cells. Results of flow cytometric determination of percentages of CD9-positive exosomes in the exosomes extracted from ADSCs by conjugation with the exosome marker CD63. (**A**) Histogram of PE-labeled CD9 flow cytometry for normal ADSCs. (**B**) Histogram of PE-labeled CD9 flow cytometry for T1DM ADSCs. (**C**) Results of quantification of the percentages of positive cells. Values shown in the graph are in the form of mean ± SEM. The number of samples is *n* = 3. Two independent evaluations were performed. Shapiro–Wilk and unpaired t-tests were used for statistical analysis. *** The *p*-value was 0.0006.

To examine the differences in the characteristics of the exosomes, we concentrated the exosomes using capture beads conjugated with CD63, which was used as an exosome marker, and a flow cytometer to evaluate the expression of CD9, which is an inflammation marker. Both CD63 and CD9 are members of the tetraspanin membrane protein family and share a four-transmembrane domain structure [14]. They have been reported to play an important role in cell membrane formation and to be expressed in the exosome membrane. CD9 knockout mice have been reported to have altered the arrangement and strength of the cell membrane and intensified inflammatory reactions [15]. A CD9-negative peak was observed in the CD63-positive exosomes extracted from the normal ADSCs (Figure 6A). In contrast, the CD63-positive exosomes extracted from the T1DM ADSCs showed no CD9-negative peaks (Figure 6B). The CD9-positive exosomes accounted for 83.13 ± 0.80% and 93.37 ± 0.24% of the exosomes extracted from the normal ADSCs and the T1DM ADSCs, respectively, showing a significantly increased proportion of CD9-positive exosomes in the latter.

## 3. Discussion

Regenerative therapy using insulin-producing cells derived from stem cells has been studied as a radical treatment approach for type 1 diabetes mellitus [16]. however, it has not been verified whether stem cells derived from patients with type 1 diabetes mellitus have the same cellular structure as stem cells derived from healthy humans. The degeneration of stem cell organelles deteriorates the quality of stem cells and affects their stemness and transplantation efficiency. In this study, we characterized organelles in T1DM ADSCs. We demonstrated that T1DM ADSCs, which are stem cells derived from patients with type 1 diabetes mellitus, degenerate differently from those derived from patients with type 2 diabetes mellitus, which was characterized by the evident enlargement of spherical vesicles. The exosomes extracted from T1DM ADSCs were found to be enlarged, reduced in amount, and contained an increased percentage of exosomes positive for the tetraspanin CD9. The findings of this study provided new insights into the structural changes of MSCs occurring in patients with type 1 diabetes mellitus.

Type 1 diabetes mellitus is a pathological condition in which insulin secretion is impaired due to the destruction of pancreatic β-cells, in contrast to type 2 diabetes mellitus, wherein genetic and environmental factors are responsible for insulin resistance and decreased insulin secretion [17]. We previously reported on the degeneration of organelles in T2DM ADSCs [12]. In T2DM ADSCs, large and flat cells with a swollen cytoplasm have been observed [12]. The additional characteristics of T2DM ADSCs included the heterogeneity of cells, low cell density, and swollen nuclei; however, no such characteristics have been found in T1DM ADSCs, which represented the focus of the present study. Furthermore, the structural degeneration of mitochondrial cristae was reported for T2DM ADSCs, but we did not observe any mitochondrial degeneration in T1DM ADSCs. These results demonstrated that T1DM ADSCs underwent the degeneration of organelles differently to T2DM ADSCs. These findings suggested that differences in pathogenesis and pathophysiological environments cause different degenerations of stem cell organelles depending on the disease, namely, type 1 and type 2 diabetes mellitus. In particular, the function of insulin-producing β-cells is more severely impaired in most patients with type 1 diabetes mellitus than in patients with type 2 diabetes mellitus, and those with β-cell insufficiencies require an exogenous insulin replacement. Moreover, the treatment of type 1 diabetes mellitus requires a long time because of the onset at a young age. Presumably, these differences in pathology and treatment underlie differences in the degeneration of stem cell organelles.

In this study, we showed that the degeneration in T1DM ADSCs is characterized by prominently enlarged spherical intracellular vesicles. Recently, such spherical intracellular vesicles have attracted attention as extracellular vesicles (EVs). EVs are classified into multiple different types, such as exosomes derived from endosomes and microvesicles derived from the plasma membrane [18]. EVs have been suggested to be involved in various physiological functions and pathogenic processes [19]. In particular, exosomes are membrane vesicles secreted by various cells and range approximately within 30–150 nm in diameter [20]. Exosomes contain various cell-derived proteins and RNAs, including endosome-derived proteins, proteins involved in intracellular transport, and proteins derived from the cellular membrane [21]. Besides their role in releasing unrequired cell contents [22], exosomes have attracted attention as a new mediator of intercellular communication for transporting lipids, proteins, RNAs, and other molecules in the body [23]. In this study, we found that the exosomes in T1DM ADSCs were enlarged, reduced in amount, and increased in the proportion of CD9-positive exosomes. In the exosome secretion process, intraluminal membrane vesicles (ILVs) are first formed through a budding-like process in endosomes formed by endocytosis [24]. Multivesicular endosomes (MVBs), each of which contains multiple ILVs, are fused with the cell membrane and release exosomes extracellularly [25]. In the exosome formation process, proteins, mRNA, and other substances are encapsulated in vesicles [26]. The exosome’s outer membrane is composed of a lipid bilayer membrane containing ceramides, whose composition differs from that of the cell membrane [27]. The degeneration of T1DM ADCS exosomes found in this study suggests a disorder in the exosome formation process. Detailed molecular mechanisms underlying exosome denaturation in T1DM ADSCs represent a subject for further study.

The ADSCs were extracted from Caucasian female adipose tissue. The BMI of the T1DM ADSC donor was 22, which is not obese. Ages ranged from 30 to 62 years, and the cells with the closest cell viability were selected from 11 donors for the study. The viability rate of normal ADSCs was 90%, and the viability rate of T1DM ADSCs was 87%. The quality and type of stem cells and the background of the stem cell donors have a great impact on the efficacy of stem cell-based regenerative therapy. MSCs are widely used for regenerative therapy, because they can be obtained from various tissues in the body, such as adipose tissue; however, a meta-analysis evaluating the efficacy of stem cell therapy against diabetes mellitus found no significant improvements in HbA1c and C-peptide, which are indicators of blood glucose level, after the transplantation of MSCs [28]. The deteriorated stem cell quality of MSCs derived from patients with type 1 diabetes mellitus, caused by exosome degeneration as revealed in this study, may be responsible for the reduced efficacy of regenerative therapy. Moving forward, we intend to conduct detailed analyses of the characteristics of MSCs during diabetes to find a way to improve the efficacy of regenerative therapy.

Several limitations of this study should be considered. First, this study revealed the degeneration of intracellular vesicles and exosomes, but it is not clear whether this degeneration occurs in stem cells from all type 1 diabetic patients. In future, we plan to proceed with the evaluation using stem cells derived from type 1 diabetic patients with different treatment periods and ages. Next, this study found that the exosomes increased in size and decreased in number, but it was not clarified how these exosome changes affected the pathophysiology of type 1 diabetes and the effect of stem cell transplantation. The elucidation of the pathophysiology of exosome degeneration is a future task.

We selected the most proliferative cells from the 11 donors and used them in this study. Therefore, this study was not able to clarify whether the severity of type 1 diabetes correlates with stem cell characteristics, such as exosome organization. In the future, we will clarify how the properties of stem cells differ depending on the severity of diabetes. In this study, we successfully found exosome degeneration in T1DM ADSCs. These results contribute to an improved understanding of changes in MSCs during type 1 diabetes mellitus and the realization of effective regenerative therapy.

## 4. Materials and Methods

### 4.1. Cell Culture for ADSCs

We used ADSCs of healthy humans and patients with type 1 diabetes mellitus that were characteristically positive for CD13, CD29, CD44, CD73, CD90, CD105, and CD166, which are stem cell markers, and negative for CD14, CD31, and CD45. The ADSCs were purchased from LONZA. The ADSCs T1DM ADSC (Lonza, Greenwood, SC, USA), The Normal ADSC (Lonza, Greenwood, SC, USA). The ADSCs were seeded at a density of 5000 cells/cm^2^ and cultured in a dedicated medium (ADSC-GM BulletKitTM, Lonza, Greenwood, SC, USA) in a 5% CO_2_ atmosphere at 37 °C. Stem cells after up to the fifth passage, which were confirmed to express stem cell markers, were used in the study.

### 4.2. Electron Microscopic Observation

A transmission electron microscope was used to observe the morphological characteristics of ADSCs and exosomes. Samples were pre-fixed in 2% glutaraldehyde and post-fixed in a 2% aqueous solution of osmium. The samples were then dehydrated with ethanol in steps and embedded in epoxy resin. An ultramicrotome was used to prepare 80–90 nm sections, which were mounted on a 200 mesh. The samples were stained with a 2% aqueous solution of uranium acetate and a lead stain solution and observed with a TEM (HITACHI H-7600, HITACHI, Tokyo, Japan).

### 4.3. Exosome Extraction

Exosomes were extracted using ExoQuick-TCTM. To 10 mL of the ADSC culture supernatant collected, 2 mL of the ExoQuick reagent was added. The resulting mixture was stirred sufficiently before the mixture was allowed to stand at 4 °C for one day. The mixture was centrifuged at 1500× *g* for 30 min to isolate exosomes as a precipitate.

### 4.4. Particle Size Measurement of the Exosomes 

The diameter of the exosome particles was measured using the dynamic light scattering (DLS) method. The exosome extract was diluted 50-fold with phosphate-buffered saline, and the resulting mixture was placed in a square cell for particle size measurement; a particle size distribution analyzer (Zetasizer Pro, Malvern Panalytical Japan, Tokyo, Japan) was used to measure the mean particle diameters of the exosomes.

### 4.5. Quantification of the Exosomes Based on Acetylcholinesterase (AChE) Activity

The exosomes were quantified using a colorimetric quantification kit (EXOCET Exosome Quantitation Kit (System Biosciences), Funakoshi, Tokyo, Japan) based on AChE activity. The extracted exosomes were mixed well with PBS, and the resulting mixture was incubated at 37 °C for 5 min to release exosomal proteins. After centrifugation at 1500× *g* for 5 min, the supernatant was collected and allowed to stand on ice. The EXOCET reaction solution, which was prepared immediately before the reaction, was mixed with the sample on a 96-well plate. The colorimetric reaction was carried out at room temperature for 20 min. A standard curve was prepared using EXOCET standard solutions. A plate reader was used to measure the absorbance at 405 nm.

### 4.6. Evaluation of the Exosome Markers CD63 and CD9

The expression of the exosome markers CD63 and CD9 was evaluated using flow cytometry. A 50 µL suspension of capture beads modified with CD63 antibodies was added to a 100 µL solution of the extracted exosome and mixed well. The resulting mixture was allowed to stand overnight at room temperature. After centrifugation at 2500× *g* for 5 min, the precipitate was collected. PE-labeled CD90 antibodies were added to the precipitate and were allowed to react at 4 °C for 60 min. After washing with the assay buffer, CD90-positive exosomes were detected using a flow cytometer (Sony SA3800, Sonybiotechnology, Tokyo, Japan).

### 4.7. Statistical Analysis

The Shapiro–Wilk tests was used to evaluate normality. IBM SPSS Statistics version 26.0 was used as the statistical analysis software (IBM, IGSCH, Hiroshima, Japan). If *p* < 0.05, it was judged not to follow the normal distribution, and if *p ≥* 0.05, it was judged to follow the normal distribution. A *t*-test was used for the significance test between the two groups. GraphPad Prism 8 was used as the statistical analysis software (GraphPad Prism, MDF, Tokyo, Japan). A *p*-value < 0.05 was judged to be statistically significant.

## Data Availability

The datasets generated and analyzed during the current study are available from the corresponding author upon reasonable request.
